# Exogenous Melatonin Mitigates Methyl Viologen-Triggered Oxidative Stress in Poplar Leaf

**DOI:** 10.3390/molecules23112852

**Published:** 2018-11-02

**Authors:** Fei Ding, Gang Wang, Shuoxin Zhang

**Affiliations:** 1 College of Forestry, Northwest A&F University, Yangling 712100, Shaanxi, China; fding@nwafu.edu.cn (F.D.); wang20180823@sina.com (G.W.); 2 Guizhou Academy of Forestry, Guiyang 550005, Guizhou, China; 3 Qinling National Forest Ecosystem Research Station, Huoditang, Ningshan 711600, Shaanxi, China

**Keywords:** melatonin, reactive oxygen species, oxidative stress, methyl viologen, proline, poplar

## Abstract

As a ubiquitous molecule, melatonin plays a crucial role in tolerance to multiple stresses in plants. In the present work, we report the role of exogenous melatonin in relieving oxidative stress induced by methyl viologen (MV) in poplar (*Populus alba* × *Populus glandulosa*) leaf. Leaf discs pretreated with melatonin exhibited increased tolerance to MV-mediated oxidative stress. It was observed that melatonin pretreatment effectively reduced membrane damage and lipid oxidation as demonstrated by decreased relative electrolyte leakage and malonaldehyde content in poplar leaf discs. Exogenous melatonin also stimulated activities of antioxidant enzymes, including superoxide dismutase (SOD), catalase (CAT), peroxidase (POD), and ascorbate peroxidase (APX), and enhanced accumulation of non-enzymatic antioxidants of AsA and GSH in leaf discs exposed to MV. In addition, pretreatment of melatonin prompted expression of genes for those antioxidant enzymes. Notably, exogenous melatonin increased expression of *P5CS*, a key gene for proline biosynthesis, under MV treatment. It was further observed that pretreatment with melatonin boosted activity of P5CS as well as accumulation of proline in leaf discs under MV-mediated oxidative stress. Collectively, this work provides evidence for the ameliorative effect of melatonin on MV-induced oxidative stress in poplar leaf.

## 1. Introduction

Being sessile, plants are constantly challenged by a variety of environmental factors, such as drought, extreme temperatures, salinity, nutrient deficiency, and excessive toxic metals. These unfavorable factors hamper plant growth and development, causing a huge loss in crop production [1]. The primary and common consequence of these environmental stresses is the excessive accumulation of reactive oxygen species (ROS), comprising superoxide anion radical (O_2_^−^), hydrogen peroxide (H_2_O_2_), and hydroxyl radicals (·OH) [2,3]. Major sites of ROS production are chloroplasts and mitochondria where the electron transport chain is impaired due to environmental stress to plants [4,5]. ROS are toxic to biological organisms, virtually damaging all macromolecules, including DNA, proteins, lipids and carbohydrates [3]. The oxidation of macromolecules results in the breakdown of normal cellular activities and ultimately leads to retarded growth, reduced fertility and hastened senescence in plants [3,6].

To cope with excessive production of ROS and increased oxidative stress, plants employ an efficient detoxifying system consisting of the coordinated action of antioxidant enzymes and non-enzymatic antioxidants [7,8]. The typical antioxidant enzymes involve superoxide dismutase (SOD), catalase (CAT), peroxidase (POD), and ascorbate peroxidase (APX), while representative non-enzymatic antioxidants include ascorbic acid, reduced glutathione and carotenoids [9]. Under stress conditions, increased activities of antioxidant enzymes and elevated levels of non-enzymatic antioxidants play crucial roles in scavenging the toxic ROS and maintaining redox homeostasis within plants [10].

In addition to antioxidants, osmolytes are central to adaptation to unfavorable growth conditions in plants. Proline is a well-known osmolyte and is conducive to stress tolerance by adjusting osmotic potential in plants. Studies have shown that besides being an osmolyte, proline has several other biological functions, including scavenging ROS, stabilizing proteins and modulating cellular redox homeostasis, in plants exposed to environmental stresses [11,12,13,14,15].

Melatonin (*N*-acetyl-5-methoxytryptamine) is a tryptophan-derived natural product and plays important roles in a plethora of biological processes in humans and animals. Since its identification in higher plants by two research groups in 1995 [16,17], melatonin has gained widespread attention among plant biologists. A large body of evidence has accumulated regarding the functions of melatonin in plants. The most notable role of melatonin is its antioxidant function. It is recognized as an antioxidant molecule, which functions either by directly scavenging ROS or indirectly improving antioxidant potential [18,19,20]. As an antioxidative molecule, melatonin is capable of scavenging free radicals, including reactive oxygen species and reactive nitrogen species [21,22,23]. Melatonin has also been proven effective in the reduction of ROS by improving activities of antioxidant enzymes and enhancing non-enzymatic antioxidants in plants under various stress conditions [24,25,26,27,28]. In addition, melatonin has a key role in preventing chlorophyll degradation and delaying senescence in plants [29,30]. In a recent report, melatonin has been found to be associated with leaf cuticle formation and thus improves drought resistance by reducing non-stomatal water loss in tomato plants [31].

Methyl viologen (MV, known as paraquat) is a non-selective herbicide that kills a wide range of broad-leaved weeds and annual grasses. Under light, MV acts in the chloroplasts by diverting electrons from photosystem I (PSI) and transferring them to dioxygen and, as a consequence, ROS are produced [32,33]. MV is often used as a stress agent in studies of oxidative stress. In this study, we investigated the role of melatonin in the mitigation of oxidative stress induced by MV in poplar leaf. We assessed the effect of melatonin pretreatment on poplar leaf discs by measuring levels of ROS, degree of lipid peroxidation, activities of antioxidant enzymes, contents of non-enzymatic antioxidants and transcript abundance of related genes. We were particularly interested to know whether exogenous melatonin exerts influence on proline metabolism in poplar leaf stressed by MY. The goal of this work was to provide evidence that exogenous melatonin reduces MV-induced oxidative stress in *Populus* species.

## 2. Results

### 2.1. Exogenous Melatonin Alleviates Oxidative Stress in Poplar Leaf Exposed to MV

**Figure 1:**
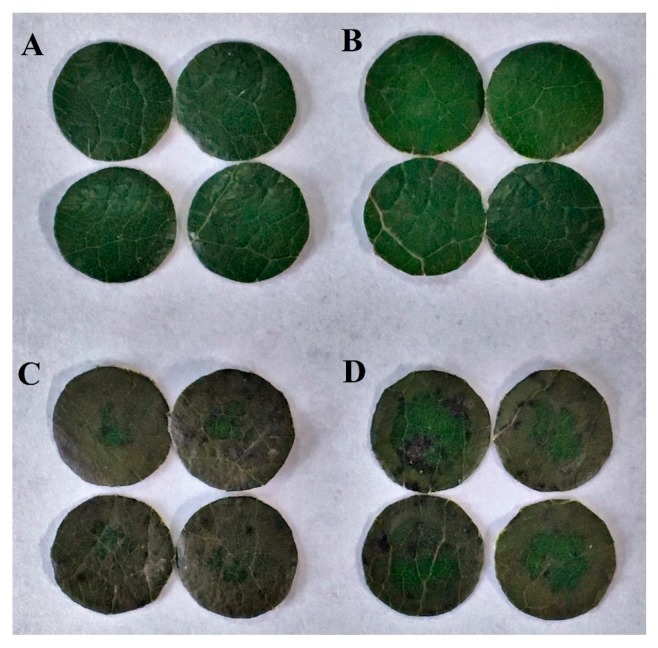
Effect of exogenous melatonin (MT) on phenotypes of poplar leaf discs exposed to methyl viologen (MV) for 24 h. (**A**) Control; (**B**) Control + MT; (**C**) MV; (**D**) MV + MT. Poplar leaf discs were pretreated with 150 μM melatonin in the dark for 12 h, and then, leaf discs were exposed to 50 μM methyl viologen in the light for 24 h.

To investigate the effect of melatonin on MV-mediated oxidative stress in poplar leaves, we first examined how melatonin pretreatment affected poplar leaf discs incubated in MV solution. Poplar leaf discs were pretreated with 150 μM melatonin in the dark for 12 h and then were incubated in 50 μM MV in the light for 24 h. After 24 h exposure to MV, leaf discs without melatonin pretreatment exhibited severe oxidative damage and evident chlorophyll loss, in contrast, leaf discs pretreated with melatonin were much less injured by MV (Figure 1), indicating the role of melatonin in the alleviation of MV-mediated oxidative stress.

### 2.2. Exogenous Melatonin Reduces MV-Mediated Accumulation of ROS in Poplar Leaf

**Figure 2:**
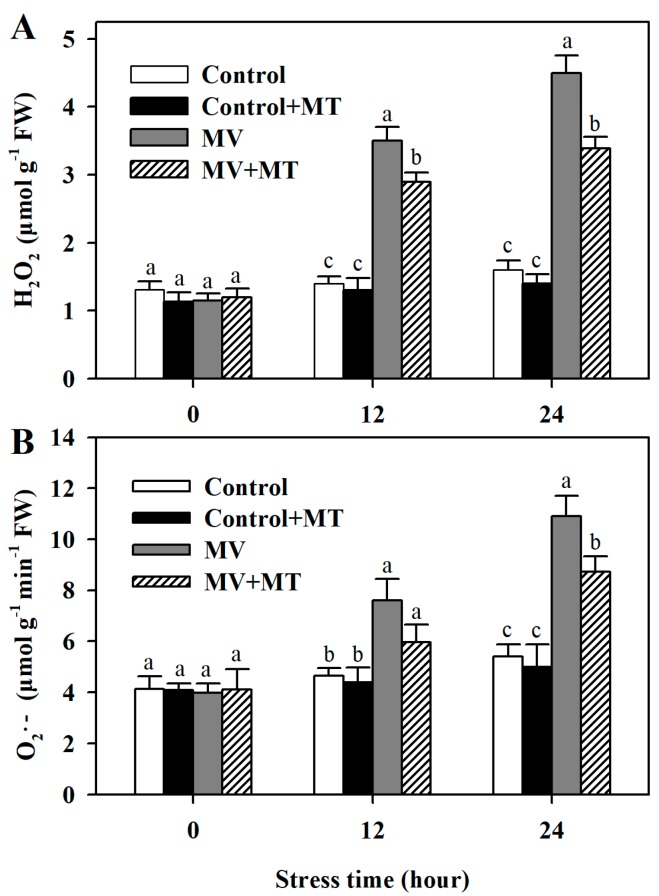
Accumulation of hydrogen peroxide and superoxide anion as affected by melatonin in poplar leaf discs exposed to MV. (**A**) H_2_O_2_ level; (**B**) O_2_^−^ level. Poplar leaf discs were pretreated with 150 μM melatonin in the dark for 12 h, and then, leaf discs were exposed to 50 μM methyl viologen in the light for 24 h. Leaf discs were collected at 0, 12 and 24 h following MV treatment. The values presented are means ± SDs (*n* = 3). Different letters indicate significant difference at *p* < 0.05 among treatments.

Following exposure to MV, significant level of H_2_O_2_ was observed in poplar leaf discs, while the addition of melatonin reduced the accumulation of H_2_O_2_. However, application of melatonin in non-MV treated discs did not have an obvious effect on the level of H_2_O_2_. Similarly, pretreatment of leaf discs with melatonin led to lower accumulation of O_2_^−^ in response to MV. These results showed that melatonin was able to decrease MV-induced accumulation of ROS in poplar leaf (Figure 2).

### 2.3. Exogenous Melatonin Alleviates MV-Triggered Oxidative Damage to Cell Membranes in Poplar Leaf

**Figure 3:**
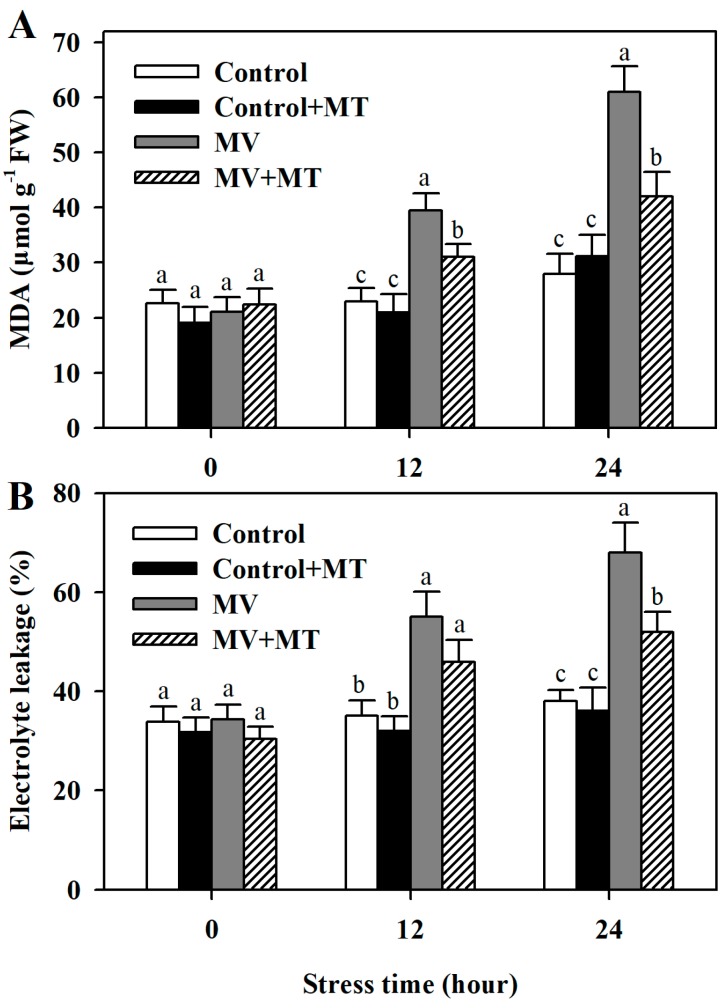
Alteration of lipid peroxidation and membrane stability as affected by melatonin in poplar leaf discs under MV stress. (**A**) MDA content; (**B**) Electrolyte leakage. Poplar leaf discs were pretreated with 150 μM melatonin in the dark for 12 h, and then, leaf discs were exposed to 50 μM methyl viologen in the light for 24 h. Leaf discs were collected at 0, 12 and 24 h following MV treatment. The values presented are means ± SDs (*n* = 3). Different letters indicate significant difference at *p* < 0.05 among treatments.

One notable detrimental effect of oxidative stress is the damage to cell membranes [3]. To determine the effect of melatonin on membrane stability in MV-treated leaf discs, we measured malonaldehyde (MDA) content and electrolyte leakage. Both MDA and electrolyte leakage were largely increased in MV-treated leaf discs compared with those in control discs, while melatonin pretreatment significantly reduced content of MDA in leaf discs under MV-triggered oxidative stress. Likewise, it was observed that pretreatment of melatonin decreased electrolyte leakage in MV-treated leaf discs (Figure 3). These results were consistent with our observation that melatonin reduces ROS accumulation in MV-treated leaf discs.

### 2.4. Exogenous Melatonin Increases Activities of Antioxidant Enzymes and Levels of Non-Enzymatic Antioxidants in Poplar Leaf Discs Exposed to MV

**Figure 4:**
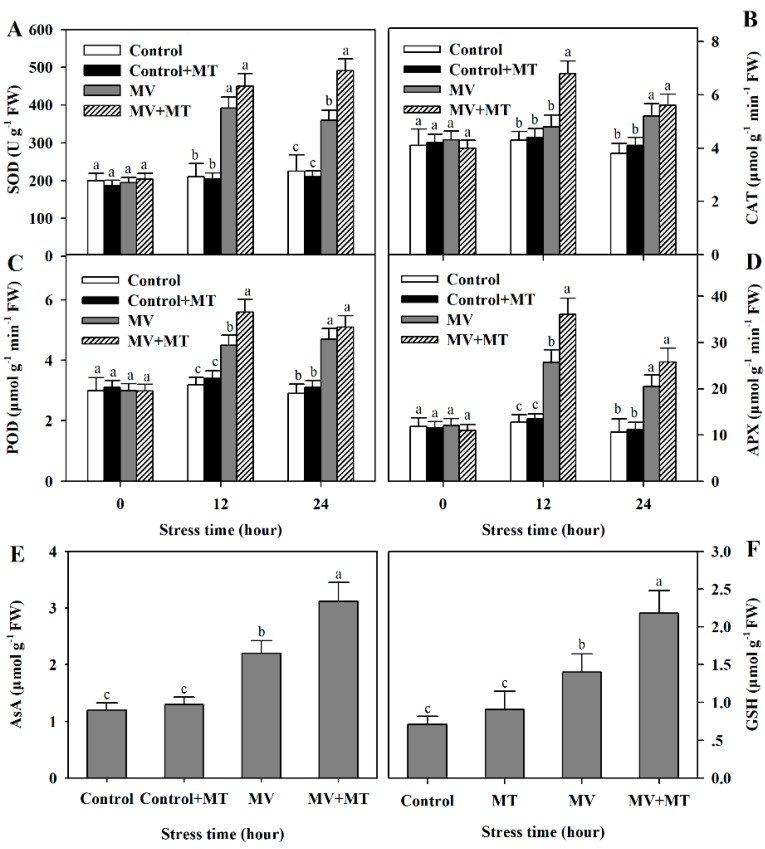
Changes in activities of antioxidant enzymes and levels of non-enzymatic antioxidants as affected by melatonin in poplar leaf discs under MV stress. (**A**) superoxide dismutase (SOD); (**B**) catalase (CAT); (**C**) peroxidase (POD); (**D**) ascorbate peroxidase (APX); (**E**) AsA. (**F**) GSH. Poplar leaf discs were pretreated with 150 μM melatonin in the dark for 12 h, and then, leaf discs were exposed to 50 μM methyl viologen in the light for 24 h. Leaf discs were collected at 0, 12 and 24 h following MV treatment. The values presented are means ± SDs (*n* = 3). Different letters indicate significant difference at *p* < 0.05 among treatments.

In the long-term evolution, plants have developed a sophisticated antioxidant system, consisting of antioxidant enzymes and non-enzymatic antioxidants, to combat oxidative stress [10]. To examine whether exogenous melatonin increased antioxidant capacity in MV-treated poplar leaf discs, we assayed activities of SOD, CAT, POD and APX, and measured the contents of antioxidants AsA and GSH. Following exposure to MV, activities of antioxidant enzymes and accumulation of non-enzymatic antioxidants were significantly induced and pretreatment of exogenous melatonin led to more pronounced enzyme activities and antioxidant accumulation in poplar leaf discs (Figure 4), indicating that exogenous melatonin positively influences antioxidant system in poplar leaf, in agreement with the observed reduction in accumulation of ROS and oxidative damage in poplar leaf.

### 2.5. Exogenous Melatonin Promotes Expression of Genes for Antioxidant Enzymes

**Figure 5:**
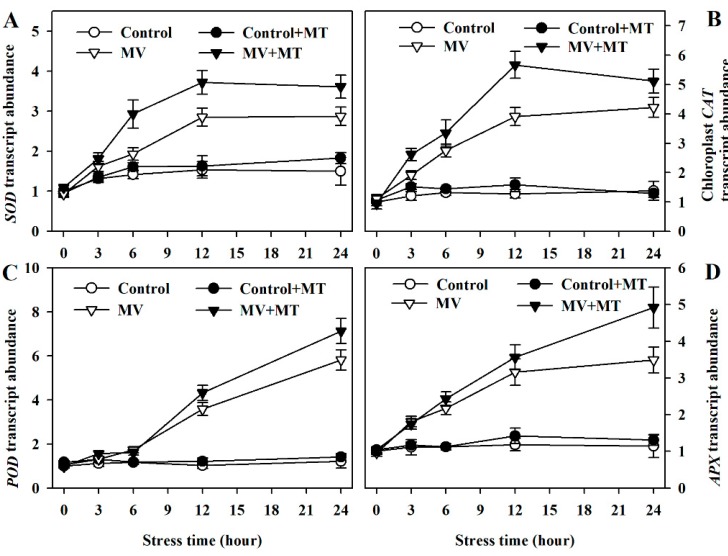
Relative transcript abundance of genes for antioxidant enzymes as affected by melatonin in poplar leaf discs under MV stress. (**A**) *Cu/Zn-SOD*; (**B**) *CAT*; (**C**) *POD*; (**D**) *Chloroplast APX*. Relative transcript abundance was determined by quantitative real-time PCR using total RNA isolated from poplar leaf discs collected at 0, 3, 6, 12 and 24 h following MV treatment. The values presented are means ± SDs (*n* = 3).

To have a better understanding of melatonin action on MV-triggered oxidative stress, we continued to investigate the expression of genes involved in the expression of antioxidant enzymes. By measuring the transcript abundance of related genes, we found that the expression of *Cu/Zn*-*SOD*, *CAT*, *POD,* and *Chloroplast APX* were increased more in melatonin-pretreated poplar leaves exposed to MV than in non-melatonin-pretreated leaves (Figure 5), suggesting the function of melatonin at the transcriptional level.

### 2.6. Exogenous Melatonin Enhances Proline Accumulation, *P5CS* Activity, and P5CS Transcript Abundance

**Figure 6:**
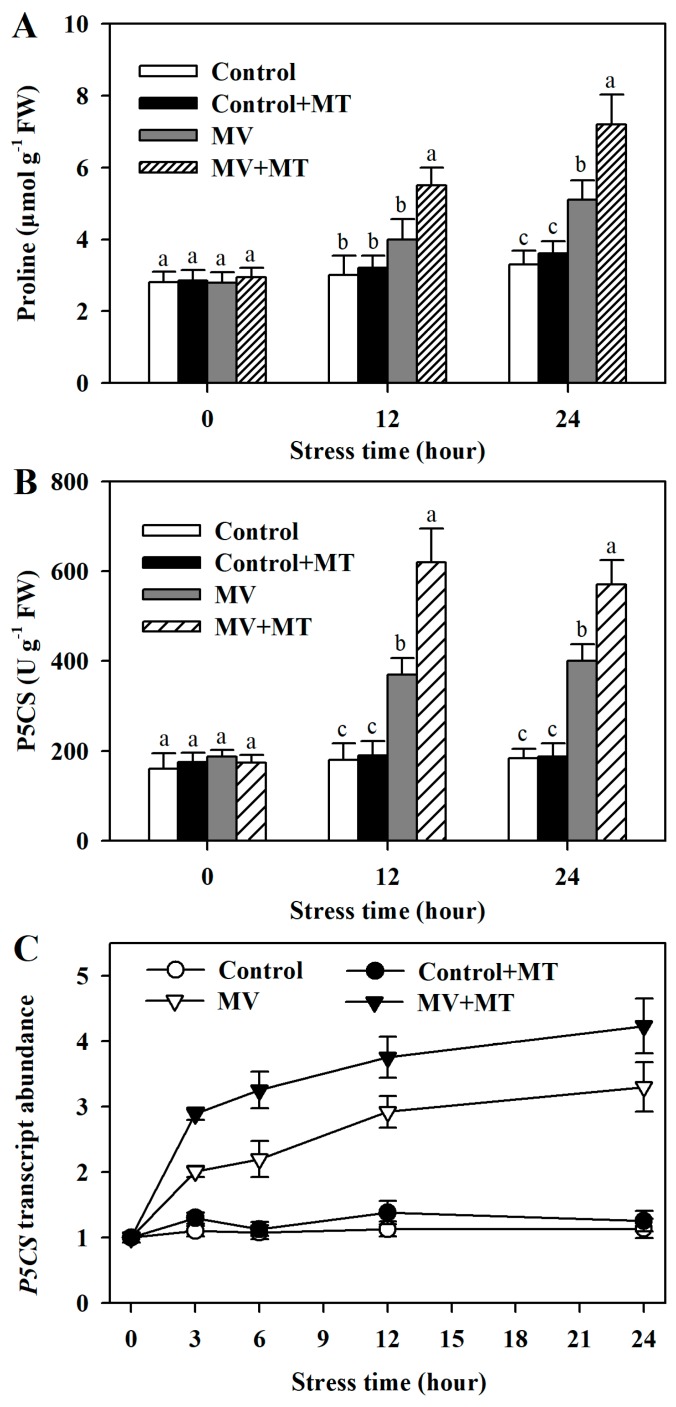
(**A**) Proline accumulation; (**B**) P5CS activity; (**C**) *P5CS* mRNA abundance as affected by melatonin in poplar leaf discs under MV stress. Poplar leaf discs were pretreated with 150 μM melatonin in the dark for 12 h, and then, leaf discs were exposed to 50 μM methyl viologen in the light for 24 h. The values presented are means ± SDs (*n* = 3). Different letters indicate significant difference at *p* < 0.05 among treatments.

Proline acts not only as an important regulator of osmotic potential but also as a scavenger of ROS [13]. Thus, we were particularly interested to know how exogenous melatonin may affect the biosynthesis of proline in poplar leaves exposed to MV. Proline accumulation was increased by MV treatment in poplar leaf discs and its accumulation was more pronounced in melatonin-pretreated leaf discs, suggestive of the regulatory role of melatonin in proline biosynthesis. Further, activity of Δ^1^-pyrroline-5-carboxylase synthase (P5CS), a key enzyme in biosynthesis of proline, was elevated in melatonin-pretreated leaf following exposure to MV. We also analyzed the transcript abundance of *P5CS*. MV treatment alone increased the expression of *P5CS*, however, melatonin pretreatment increased the expression of *P5CS* to the larger extent (Figure 6).

## 3. Discussion

ROS are important signaling molecules for activating a series of physiological response in plants. However, under environmental stress, normal metabolic activities are perturbed and generation of ROS is accelerated, leading to excessive accumulation of ROS and disruption of redox balance in plant cells [6]. High level of ROS results in oxidative stress, causing damage to cell membranes, nucleic acids, proteins and lipids in plants [3]. To cope with oxidative damage, plants have evolved an efficient ROS scavenging system, which includes antioxidant enzymes and non-enzymatic antioxidants. Plants rely heavily on this system to sustain an appropriate level of ROS under stress conditions. Melatonin, a ubiquitous and extensively studied molecule, has been demonstrated to protect plants against diverse abiotic stress factors, such as cold, heat, drought, salinity, sodic alkaline and heavy metal toxicity [27,31,34,35,36,37,38]. In these cases, one of the key functions of melatonin is to reduce stress-induced ROS in plants. In the present work, methyl viologen was used as a stress agent to investigate the role of melatonin in the mitigation of oxidative stress in poplar leaf. We have concluded that melatonin is involved in the protection of poplar leaf against MV-mediated oxidative stress and the evidence leading to this conclusion includes (1) pretreatment of polar leaf with melatonin alleviated MV-induced lipid peroxidation and decreased electrolyte leakage; (2) exogenous melatonin increased activities of antioxidant enzymes, including SOD, CAT, POD and APX, and promoted the expression of genes for these enzymes; (3) exogenous application of melatonin enhanced the accumulation of proline, which acts both as an osmolyte and a ROS scavenger; (4) exogenous melatonin increased the activity of P5CS, a key enzyme in proline biosynthesis and increased transcript abundance of *P5CS* under MV-mediated oxidative stress.

There is growing evidence that melatonin is a potent anti-oxidative molecule, which acts by improving antioxidant potential in plants [19,26,39]. In this study, we observed that MV-induced oxidation caused damage to poplar leaf discs, whereas melatonin obviously reduced the damage, indicative of the anti-oxidative role of melatonin in poplar leaf. This observation is consistent with a recent study that melatonin decreases MV-induced damage to apple leaf [40]. In the light, MV diverts electrons from Photosystem I to oxygen, giving rise to ROS [32], which is also confirmed by our results. In the current work, exposure of poplar leaf discs to MV aggravated the accumulation of superoxide anion radical and hydrogen peroxide compared with exposure of leaf discs to water control, while pretreatment of polar leaf with melatonin markedly suppressed the production of these two ROS. There results further validate that melatonin might act as a strong antioxidant.

Oxidative stress causes lipid peroxidation and damages membranes [3]. We found that MV treatment led to a significant rise in the level of MDA, suggestive of severe lipid oxidation. Previous studies have shown that melatonin maintains membrane stability by reducing lipid oxidation in plants under stress conditions [26,39]. Our results showed that exogenous melatonin application prior to exposure of poplar leaf discs to MV significantly reduced lipid peroxidation as indicated by relatively low level of MDA. Electrolyte leakage is generally considered as a reliable indicator of cell membrane integrity. We thus measured electrolyte leakage of poplar leaf discs in different treatments. MV treatment caused a dramatic increase in electrolyte leakage in poplar leaf discs, while melatonin pretreatment decreased electrolyte leakage. These results suggest the involvement of melatonin in reducing lipid peroxidation and maintaining membrane integrity.

The antioxidant system, comprising antioxidant enzymes and non-enzymatic oxidants, plays an essential role in adaptation of plants to environmental stresses by scavenging ROS [8]. The reductions in ROS are dependent on the activation of the antioxidant system in plants. Previous studies have established that melatonin promotes activities of antioxidant enzymes and accumulation of non-enzymatic antioxidants in plants under various environmental stress conditions [24,26,35,41]. Consistently, our study showed that melatonin application to poplar leaf discs before MV treatment substantially stimulated activities of SOD, CAT, POD and APX. In addition, non-enzymatic antioxidants, such as AsA and GSH, were markedly increased in melatonin-pretreated leaf discs under MV-induced oxidative stress. Our results further demonstrate that melatonin functions to enhance the antioxidant capacity of plants under stress conditions.

Melatonin is also recognized as a molecule that contributes to antioxidant capacity by prompting gene expression [34,42]. Our results showed that in response to MV, transcript abundance was increased in *SOD*, *CAT*, *POD* and chloroplast *APX*, and pretreatment with melatonin led to even higher transcript level of these genes. Likewise, the expression of these genes is enhanced by application of melatonin in other species, such as tomato, bermudagrass and apple [26,37,39,40]. We therefore infer from these results that melatonin acts in multiple ways to quench ROS. It may work as a direct antioxidant molecule as well as an indirect inducer of gene expression. However, as an indirect antioxidant molecule, the underlying molecular mechanism of stimulating gene expression is still largely unknown.

In response to environmental stress, plants tend to accumulate compatible solutes, such as sugars, betaines, and proline, to adjust osmotic potential. In this study, we observed a pronounced increase in proline in leaf discs exposed to MV, suggesting that in addition to being an osmolyte, proline may act as an antioxidant and thus plays a role in protecting plants against oxidative stress. Proline has been reported as a ROS scavenger in a previous study, in which it was found that proline was able to protect yeast cells from ROS-generating herbicide methyl viologen [43]. However, it needs to be noted that it is still debated that proline works as a direct ROS quencher [44]. It is interesting to observe that the increase in proline was more dramatic in leaf discs pretreated with melatonin prior to MV treatment, indicating that melatonin mediates the biosynthesis of proline during oxidative stress. To uncover the role of melatonin in the biosynthesis of proline, we analyzed the activity of P5CS, a key enzyme responsible for proline biosynthesis. It was shown that MV treatment increased P5CS activity and addition of melatonin before MV treatment further stimulated P5CS activity. Furthermore, transcript abundance of *P5CS* was greatly increased by exogenous application of melatonin to polar leaf under MV stress. The elevated P5CS activity and gene expression suggest that melatonin is, at least in part, attributed to the increased accumulation of proline under MV-mediated oxidative stress. However, due to limited data in the present study, the exact mechanism of melatonin modulating P5CS activity and *P5CS* expression is still unknown and needs further investigation.

## 4. Materials and Methods

### 4.1. Plant Materials and Treatment

Plant materials were sampled from poplar (*Populus alba* × *Populus glandulosa*) grown at Northwest A&F University (34°200′ N, 108°240′ E). Current-year branches were cut and transported to the laboratory in a black plastic bag. Fully expanded leaves were detached and cleaned with distilled water. Then, leaf discs (diameter 1.0 cm) were punched with a cork borer from leaves avoiding the mid-rib and main veins. A total of 360 leaf discs were collected and randomly placed into 12 petri dishes, with each containing 30 discs. The concentrations of melatonin (150 μM) and MV (50 μM) were selected based on a preliminary study (Figures S1 and S2). Six petri dishes were filled with 20 mL distilled water and the other six dishes were filled with 20 mL 150 μM melatonin solution. Then, all petri dishes were maintained in the dark at 25 °C for 12 h. Following that, leaf discs from each petri dish were collected and dried quickly with paper towel, and were placed back into emptied dishes. Three of six petri dishes that has been filled with water in the dark were filled with 20 mL distilled water again and the three petri dishes were filled with 20 mL 50 μM methyl viologen (Sigma-Aldrich, St. Louis, MO, USA). Three of six petri dishes that has been filled with melatonin solution in the dark were filled with 20 mL distilled water and three petri dishes were filled with 20 mL 50 μM methyl viologen. As a result, there were four different groups of leaf discs: (1) Control: leaf disks were first incubated with water in the dark, then were again in water in the light; (2) Control + MT: leaf disks were first incubated with melatonin solution in the dark, then were in water in the light; (3) MV: leaf disks were first incubated with water in the dark, then were in MV solution in the light; (4) MV + MT: leaf disks were first incubated with melatonin solution in the dark, then were in MV solution in the light. All petri dishes were maintained in the light of 200 μmol m^−2^·s^−1^ at 25 °C for 24 h in a growth chamber.

### 4.2. Measurement of ROS Accumulation

Leaf discs from different treatments were collected at 0, 12 and 24 h after petri dishes were placed in the light to determine the accumulation of H_2_O_2_ and O_2_^−^. The H_2_O_2_ was extracted with 5% (*w*/*v*) trichloroacetic acid and measured by monitoring the absorbance of titanium-peroxide complex at 410 nm as described previously [45]. O_2_^−^ was detected with nitroblue tetrazolium (NBT) method as described in a previous study [46].

### 4.3. Measurement of Malonaldehyde (MDA) Content

Leaf discs from different treatments were collected at 0, 12 and 24 h after petri dishes were placed in the light to determine MDA content. MDA level was measured according to a previous study [47]. MDA was extracted with trichloroacetic acid and assessed using thiobarbituric acid, and was quantified by measuring the absorbance of the supernatant at 450, 532, and 600 nm.

### 4.4. Measurement of Electrolyte Leakage

Leaf discs from different treatments were collected at 0, 12 and 24 h after petri dishes were placed in the light to determine the electrolyte leakage. Electrolyte leakage was measured as described in a previous study [48].

### 4.5. Measurement of Antioxidant Enzyme Activities

Poplar leaf discs (0.1 g) were ground in a mortar with 50 mM potassium phosphate buffer (pH 7.0) containing 0.1 mM EDTA and 1% polyvinylpyrrolidone (*w*/*v*). The extraction was centrifuged and the supernatant was used for the determination of activities of SOD, CAT, POD, and APX as described in previous studies [49,50,51].

### 4.6. Measurement of AsA and GSH

Leaf discs from different treatments were collected at 0, 12 and 24 h after petri dishes were placed in the light to determine the content of AsA (Ascorbate acid) and GSH (glutathione). The content of AsA was measured as described previously [52]. AsA was extracted by grinding 0.1 g leaf samples in 6% (*v*/*v*) cold HClO_4_. After centrifuging the crude extract, the supernatant was collected for further analysis. AsA content was measured by determining the absorbance difference of the supernatant at 265 nm in 200 mM sodium acetate buffer (pH 5.6) before and after 15 min incubation with 1.5 units of AsA oxidase.

GSH was measured according to a previous study [53]. GSH was extracted by homogenizing 0.1 g leaf discs in 5% sulfosalicylic acid and then crude extraction was centrifuged at 14,000 g for 10 min at 4 °C. For measurement of GSH, the supernatant was mixed with buffers and 5,5′-dithiobis-(2-nitrobenzoic acid) (DTNB) was added. The increase in absorbance at 412 nm was used for calculating the content of GSH.

### 4.7. Measurement of Proline

Leaf discs from different treatments were collected at 24 h after petri dishes were placed in the light to determine the proline content. The proline content was determined according to a previous study [54]. Briefly, leaf discs were ground in 7.5 mL 3% sulfosalicylic acid and filtered. Then, the mixture of 2 mL filtrate, 2 mL of ninhydrin reagent and 2 mL glacial acetic acid was heated at 100 °C for 1 h and then placed on ice for 20 min. The mixture was extracted in 4 mL of toluene. The absorbance at 520 nm of the extract was used to calculate the content of proline.

### 4.8. Measurement of P5CS Activity

The activity of ∆^1^-pyrroline-5-carboxylase synthase (P5CS) was determined as described previously [55]. P5CS was measured as the rate of consumption of NADPH, which was monitored as decrease in absorption at 340 nm as a function of time.

### 4.9. Measurement of Transcript Abundance by Quantitative Real-Time PCR

Leaf discs were collected at 0 h, 3 h, 6 h, 12 h and 14 h in different treatments for gene expression analysis. Total RNA was extracted from leaf discs and was used for cDNA synthesis. Quantitative real-time PCR was performed to determine transcript abundance using SYBR^®^ Premix Ex TaqTM (TaKaRa, Dalian, China) according to manufacturer’s instructions. Each real-time PCR reaction was performed on iQ5 Multicolor Real-Time PCR Detection System (BIO-RAD, Hercules, CA, USA) and the program is 1 cycle of 30 s at 95 °C, followed by 40 cycles of 5 s at 95 °C, 30 s at 60 °C. All primers in this study are listed in Table 1.

### 4.10. Statistical Analysis

All experiments in this study were repeated at least three times, and the values presented are the means ± SDs. Duncan’s multiple range test was performed to compare the difference among treatments. Different letters in figures indicate significant difference at *p* < 0.05.

## 5. Conclusions

Our study has demonstrated the ameliorative effect of exogenous melatonin on MV-mediated oxidative stress in poplar leaf. Exogenous melatonin reduces MV-induced accumulation of ROS and maintains membrane integrity by enhancing antioxidant capacity of poplar leaf. Melatonin improves antioxidant capacity by stimulating activities of antioxidant enzymes, increasing transcript abundance of related genes, and enhancing accumulation of non-enzymatic antioxidants. Particularly, exogenous melatonin promotes accumulation of proline, a well-known osmolyte, by improving P5CS activity and increasing *P5CS* expression in poplar leaf under MV-induced oxidative stress. 

## Figures and Tables

**Table 1:** Primers used in RT-PCR.

Gene	Primer Sequence (5′–3′)
*Actin*	F: AGGGCAGTGTTTCCAAGTATTG
	R: GTGTGATGCCAAATCTTCTCCAT
*CAT*	F: ATGGATCCTTACAAGCATCGTT
	R: GCACTAGCACCTCTAGCATGAAT
*Chloroplast APX*	F: ATGGCTTCTTTAACTATCCTCGG
	R: TTGGCCCGAAATTTGACAGTT
*P5CS*	F: AGTCTCGACCAGATGCACTAGT
	R: CTAGCCCAATAAGTCTTCCACC
*POD*	F: ATGGGATTAGGGTACTGCACG
	R: AGATATAAGGGTCTGCCTCTGG
*Cu/Zn-SOD*	F: AGCTTCAAGCTTCATTTCCGTG
	R: TAGCGCTCTGCAATTTGACAAC

## Supplementary Materials

The following are available online. Figure S1: Oxidative effects of different concentrations of methyl viologen (MV) on poplar leaf discs, Figure S2: Selection of proper melatonin (MT) concentration.
