# Supplementary material for: Exogenous Melatonin Mitigates Methyl Viologen-Triggered Oxidative Stress in Poplar Leaf
Source: Molecules. 2018 Nov 2;23(11):2852. doi: 10.3390/molecules23112852 (PMC6278511; doi:10.3390/molecules23112852)
Supplement: Supplementary file 1 [file molecules-23-02852-s001.pdf]

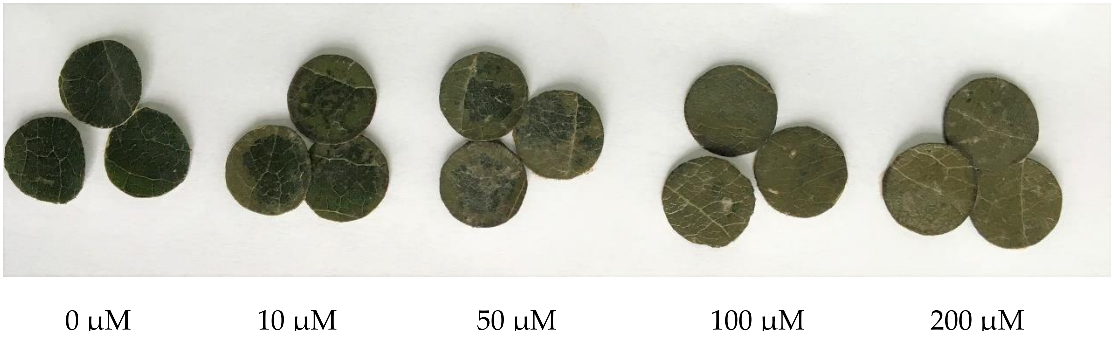

Figure S1. Oxidative effects of different concentrations of methyl viologen (MV) on poplar leaf discs. Leaf discs were incubated in MV solutions in the light for 24 h. Based on the symptoms, 50 µM MV was used for further study.

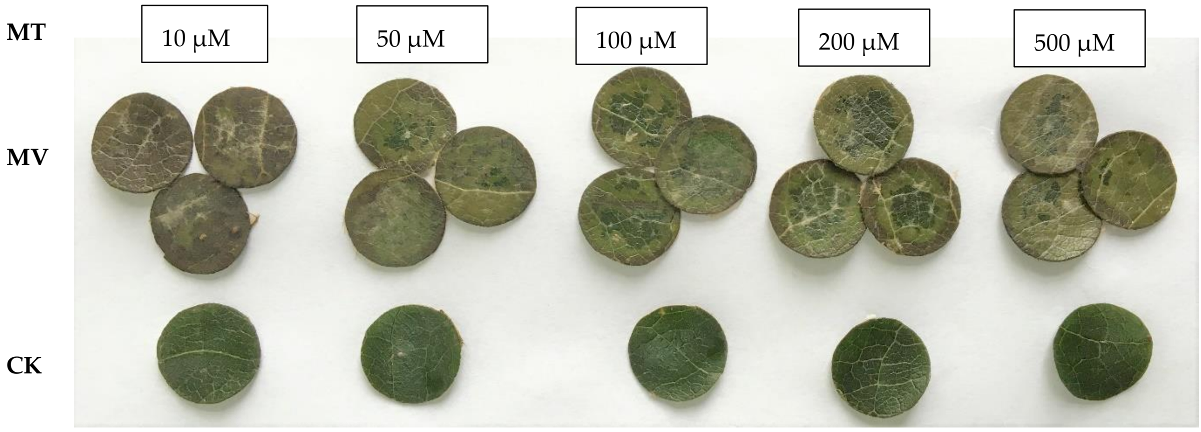

Figure S2. Selection of proper melatonin (MT) concentration. Leaf discs were first incubated with different concentrations of melatonin in the dark for 12 h and then were treated with 50 µM MV for 30 h in the light. Based on the symptoms, 150 µM melatonin was used for analysis in this study.
